# 

**Published:** 1863-07

**Authors:** 


### CONTENTS.

| ART. | | PAGE |
|---|---|---|
| I. | Ghosts, Patent and Prescriptive . . . . . . . . . . . . | 393 |
| II. | On the Law of Certainty. By the Rev. W. G. Davies . . . | 432 |
| III. | On Mental and Physical Life in Relation to Time . . . . . | 459 |
| IV. | Observations on some of the Operations of the Human Understanding. By John Alexander Davies . . . . . . . . . | 467 |
| V. | Comparative Medicine . . . . . . . . . . . . . . . . | 489 |
| VI. | Physiognomy of the Insane. By Dr. Laurent . . . . . . | 495 |
| VII. | Sensation Novels . . . . . . . . . . . . . . . . | 513 |
| VIII. | The Discovery and Discoverer of Etherization . . . . . . | 519 |
| IX. | The State of Lunacy in Scotland . . . . . . . . . . . | 534 |
| X. | On "Spiritualism" as a Cause of Insanity. By M. Burlet . | 542 |
| XI. | Gossip . . . . . . . . . . . . . . . . . . | 554 |
| XII. | Literary Retrospect . . . . . . . . . . . . . . | 563 |
| | Foreign Medico-Psychological Literature: | |
| | 1. Colonization of the Insane . . . . . . . . . . | 576 |
| | 2. Suicide in Bavaria . . . . . . . . . . . . . | 579 |
| | 3. Muscular Hallucinations . . . . . . . . . . . | 580 |

No. XII. will be published on the 1st of October.

